# T_1_-weighted *in vivo* human whole brain MRI dataset with an ultrahigh isotropic resolution of 250 μm

**DOI:** 10.1038/sdata.2017.32

**Published:** 2017-03-14

**Authors:** Falk Lüsebrink, Alessandro Sciarra, Hendrik Mattern, Renat Yakupov, Oliver Speck

**Affiliations:** 1 Department of Biomedical Magnetic Resonance, Otto-von-Guericke University, 39120 Magdeburg, Germany; 2 Center for Behavioral Brain Sciences, 39118 Magdeburg, Germany; 3 Leibnitz Institute for Neurobiology, 39118 Magdeburg, Germany; 4 German Center for Neurodegenerative Disease (DZNE), site 39120 Magdeburg, Germany

**Keywords:** Brain imaging, Brain, Imaging techniques, Magnetic resonance imaging

## Abstract

We present an ultrahigh resolution *in vivo* human brain magnetic resonance imaging (MRI) dataset. It consists of T_1_-weighted whole brain anatomical data acquired at 7 Tesla with a nominal isotropic resolution of 250 μm of a single young healthy Caucasian subject and was recorded using prospective motion correction. The raw data amounts to approximately 1.2 TB and was acquired in eight hours total scan time. The resolution of this dataset is far beyond any previously published *in vivo* structural whole brain dataset. Its potential use is to build an *in vivo* MR brain atlas. Methods for image reconstruction and image restoration can be improved as the raw data is made available. Pre-processing and segmentation procedures can possibly be enhanced for high magnetic field strength and ultrahigh resolution data. Furthermore, potential resolution induced changes in quantitative data analysis can be assessed, e.g., cortical thickness or volumetric measures, as high quality images with an isotropic resolution of 1 and 0.5 mm of the same subject are included in the repository as well.

## Background & Summary

**Figure 1:**
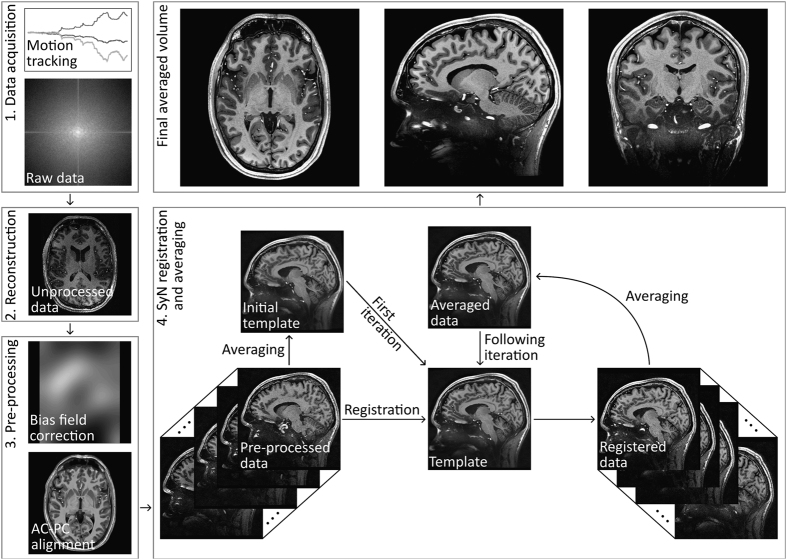
Flow chart of registration and averaging procedure. Prior to the raw data acquisition by using prospective motion correction the field of view is repositioned per k-space line excitation to remediate subject motion. After reconstruction, the data is bias field corrected and aligned to a common space. The pre-processed data is averaged and used as an initial template for the SyN registration^26^. Subsequently, the registered data is averaged and used as a template for the following registration iterations. A total number of four iterations have been run to create the final averaged volume.

Bridging gaps between optical microscopy of histological specimens and non-invasive *in vivo* imaging is one major goal of ultrahigh field magnetic resonance imaging. MRI offers insight into the development of the human brain by examination of structural changes. Quantifying these changes of the brain allows investigation of age-related and pathological processes, e.g., neurodegenerative or psychiatric disorders. However, most studies acquire T_1_-weighted anatomical data at an isotropic resolution of approximately 1 mm and yet try to identify sub-millimeter variations^1^. Recently, human MR scanners with higher field strength of 7 Tesla or more became more widespread. Higher field strength allows greater signal-to-noise ratio (SNR) (ref. 2) and, in return, enables increased spatial resolution. Increased spatial resolution of anatomical data may lead to superior quantification results^3^, e.g., due to decreased partial volume effects and therefore potentially more precise segmentations. However, high spatial resolution imaging with sufficient SNR leads to prolonged time of acquisition. Even well-versed subjects tend to move a few millimeters during an hour of scan time, limiting the effective resolution for *in vivo* imaging. Utilizing supremely accurate prospective motion correction techniques even microscopic motion can be corrected during image acquisition rendering ultrahigh resolution *in vivo* imaging possible^4^. Here, we utilized an in-bore optical tracking system with a single marker adhered to an individually manufactured mouthpiece to remediate involuntary subject motion and physiologically induced rigid motion of the head, e.g., due to heart beat and respiration. Our imaging protocol has been motivated by the goal to acquire T_1_-weighted whole brain anatomical data with a nominal isotropic resolution of 250 μm, maximized SNR, and a tolerable scan time of one hour per volume (Table 1). The resolution is far beyond any previously published *in vivo* structural whole brain dataset resulting in a voxel volume that is 64 times smaller compared to the standard clinical resolution of 1 mm and would require 64^2^ times longer scan time to result in identical SNR with traditional methods. To achieve acceptable SNR we used a 7 T MR system and acquired eight T_1_-weighted volumes of a single young healthy Caucasian subject using prospective motion correction. After inhomogeneity correction and reorientation of the volumes to a common space, they were averaged to increase SNR (Fig. 1).

**Figure 2:**
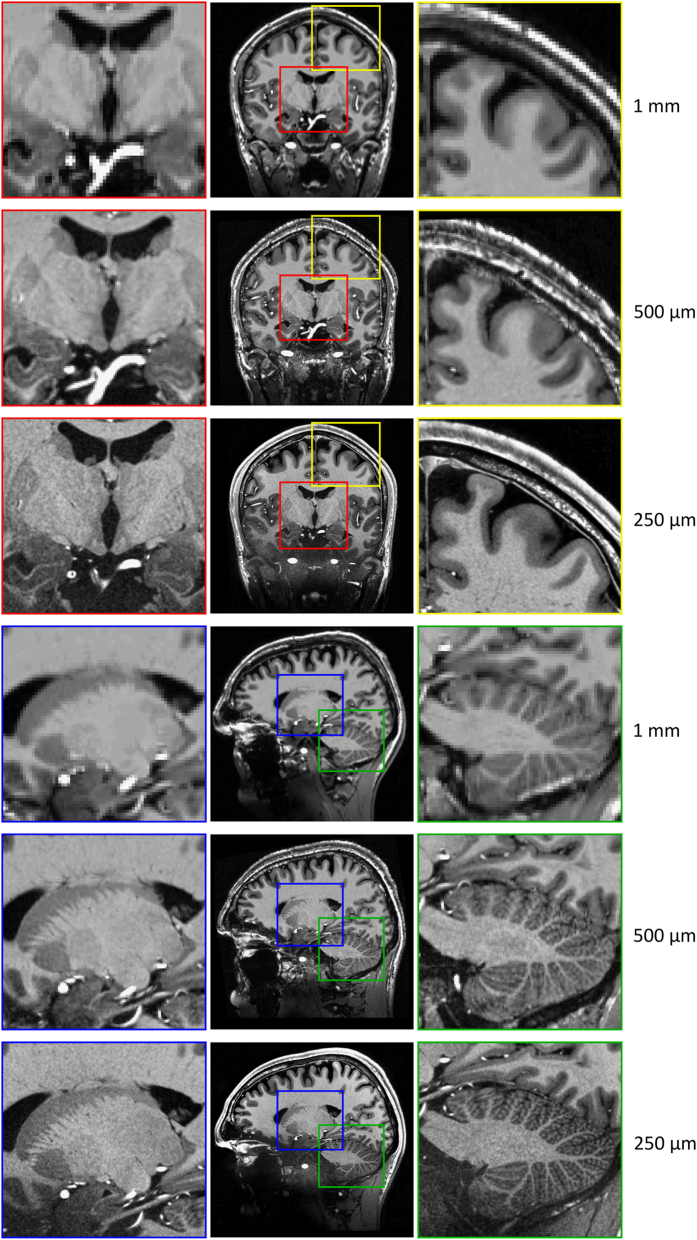
Coronal and sagittal view of isotropic 1 mm, 500 μm, and 250 μm data. The red box displays among other structures the mammillary body, the amygdala, and the hippocampus which can be better delimited with increasing resolution. The amygdala and hippocampus are separated by a thin white matter band, the amygdalo-hippocampal border (AHB). It can be depicted more precisely at higher resolution^31^ and is clearly visible despite the SNR drop in the temporal lobes due to radiofrequency (RF)-field inhomogeneity. In the yellow box the increased detail to visualize the Dura mater is illustrated; at a resolution of 250 μm it can be seen sharply. The blue box visualizes the striate bodies at great detail and enables delineation of subcortical structures, e.g., Pulvinar, Caudate, or Putamen. The green box visualizes the cerebellum at great detail, enabling depiction of its folia.

The resolution was chosen to investigate the limitation of the imaging and tracking system and to potentially illustrate anatomical structures *in vivo* for the first time or in much greater detail (Fig. 2). For example, the amygdala and hippocampal volume amounts to approximately 1.25 ml (ref. 5) and 3.5 ml (ref. 6), respectively. At an isotropic voxel size of 1 mm this relates to a cube with an edge length of 11 and accordingly 15 voxels only. As human brain atlases based on *in vivo* data are usually on a macroscopic resolution of 1 mm only^7^, our dataset could potentially assist in the segmentation of brain structural subfields, e.g., hippocampus and thalamus. At present, the highest resolution brain model has been published by Amunts *et al.*^8^ with an isotropic resolution of 20 μm derived of a post mortem brain and its histological cuts. However, transferring conclusion from post mortem to *in vivo* is difficult due to non-linear shrinkage artifacts during the fixation process^9,10^. To our knowledge, the highest resolution *in vivo* data acquired thus far published^11^ consists of T_1_-weighted, T_2_*-weighted, and turbo spin echo (TSE) data with a nominal isotropic resolution of 350 to 380 μm and is publicly available. However, raw data is not included.

The presented dataset (Data Citation 1) could be used to improve methods for image reconstruction and image restoration with low SNR. Furthermore, pre-processing and segmentation procedures could be enhanced for high field strength and ultrahigh resolution data. It allows conducting studies to identify potential resolution induced changes in quantitative data analysis by cortical thickness estimation or volumetric measures. In the near future the repository shall be extended by data of more subjects to depict norm variations. Additionally, data of the same subject is planned to be extended with repeat scans on an annual basis to enable longitudinal processing, while exploiting the newest advances in image acquisition methods available at our site. Even ultrahigh resolution data of different contrasts, such as T_2_-, T_2_*-weighting or quantitative susceptibility mapping (QSM) could be added for additional information.

## Methods

### Participant

One male Caucasian subject (born 1982) with no known history of neurological disorders participated in this study. He has been subject in a few hundred MR experiments at 3 and 7 Tesla in various studies (structural, fMRI, DTI, SWI, ToF, etc.) and is known to move very little even during extensive scanning sessions of up to two and half hours. He was fully instructed about the nature of the study and gave written informed consent for participation in the study as well as for publicly sharing all obtained data. The study was approved by the ethics committee of the Otto-von-Guericke University, Magdeburg, Germany.

### Data acquisition

Scanning has been conducted on a whole body 7 T MR scanner (Siemens Healthcare, Erlangen, Germany) using a 32-channel receive head coil (Nova Medical Head Coil, Siemens Healthcare, Erlangen, Germany). The vendor’s product implementation of the MPRAGE sequence^12^ was used to acquire the image data. It has been extended to allow a resolution of up to 2,048 instead of 512, acquisition of up to 1,024 partitions, and to facilitate prospective motion correction by adjusting the field of view (FoV) in real-time^13^. All images within the repository have been acquired using this sequence and prospective motion correction.

The imaging protocol is briefly described in Table 1. The entire protocol has been included in the repository for further details. Parameters were chosen with the goal to acquire a 250 μm MPRAGE dataset with full brain coverage. Eight single volumes have been acquired in five sessions over a period of three months. In the first session an additional PD-weighted volume with an isotropic resolution of 250 μm was acquired. In the other sessions two MPRAGE volumes were acquired in succession. However, during the third session the scanner aborted the measurement close to the end due to a heating issue, not related to SAR exposure, irrecoverably losing the data. No acceleration techniques, e.g., GRAPPA, have been used in acquiring any of the images. Slice partial Fourier had to be set to 6/8, along with a relatively high bandwidth of 440 Hertz per pixel to achieve the required inversion time (TI) for good contrast between grey and white matter. However, the high bandwidth reduces the SNR compared to lower bandwidth acquisitions by the square root of its ratio.

The extremely high spatial resolution of the dataset acquired at 7 T limits parameter choices of the sequence. In conjunction with the commercially available 32-channel head coil this results in signal voids due to (RF)-field inhomogeneity and very low SNR in some brain regions, e.g., deep brain and temporal lobes and can only be corrected partially by averaging the data. Using for example more advanced (custom build) coils or parallel transmit may allow acquiring more homogenous data. Facilitating 2D-GRAPPA (ref. 14) in slice encoding direction should potentially allow reducing the bandwidth and TI to increase SNR in general and improve contrast-to-noise ratio (CNR) of GM/WM (ref. 15), respectively. Additionally, application of specially designed magnetization inversion pulses for ultrahigh field such as the TR-FOCI (ref. 16) may increase the SNR and contrast in those regions in future acquisitions. Furthermore, this pulse reduces the susceptibility artifact superior to the sphenoidal sinuses and medial to the frontal sinus, which causes hyperintense areas in the inferior frontal lobe and anterior prefrontal lobe, respectively.

### Motion correction setup

This is a brief description of the hardware setup only. Detailed information regarding our experimental setup can be found in Stucht *et al.*^4^ and for an in-depth explanation of the hardware and validation we refer to Maclaren *et al.*^17^.

**Figure 3:**
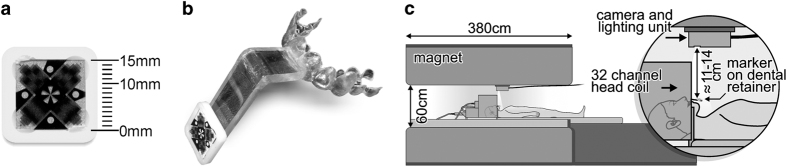
Setup for prospective motion correction using optical Moiré phase tracking. (**a**) Moiré phase tracking marker. (**b**) Individually manufactured mouthpiece with extension to protrude from the head coil. (**c**) Experimental setup for motion tracking during the MRI measurements. Image courtesy to Stucht *et al.*^4^

The subjects’ motion is optically tracked with 80 frames per second utilizing an MR-compatible camera (MT384i, Metria Innovation Inc., Milwaukee, WI, USA) and one marker with Moiré patterns (Fig. 3a). The camera is mounted above the head coil on the upper side of the scanner’s bore facing directly downwards onto the subject’s neck. The tracking marker is adhered to an extension of an individually manufactured mouthpiece of the six central upper jaw’s teeth (Fig. 3b). Utilizing this setup motion can be tracked along six degrees of freedom with high tracking precision of up to 10 μm and 0.01 degrees^17^. By attaching the marker to the extension of the mouthpiece it protrudes from the helmet-design 32-channel head coil while being rigidly fixated to the subject’s head. The line of sight cannot be lost and the observed motion represents the rigid motion of the skull and thus brain accurately. Non-rigid motion, for example due to pulsation of the vascular or ventricular system, cannot be corrected using this method but seems to be very minor as demonstrated by the high level of detail even at the high resolution acquired here. The scanner’s FoV is updated prior to excitation of each k-space line according to the marker’s position by recalculation of the imaging gradients and frequencies.

### Data reconstruction

Reconstruction of the raw data was performed on the vendor’s recent MARS reconstruction computer and was stored in DICOM format. The exact details of the reconstruction pipeline is not made publicly available by the vendor, however, fundamentally consists of zero-filling the missing k-space data in case of partial Fourier acquisition, afterwards fast Fourier transformation, and the vendor’s implementation of ‘Adaptive Combination’^18^ to combine each receive channel’s complex data into the final image volume. The slice partial Fourier acquisition of 6/8 and its reconstruction by zero-filling leads to an increase of approximately 15% in the full width half maximum (FWHM) of the point spread function^11^, degrading the effective resolution to ≈290 μm instead of 250 μm in the slice encoding direction (L-R).

Distortion correction has not been applied during the reconstruction process to avoid any processing step that reduces the originality of the data and to ensure reproducibility. The vendor does not allow publicly sharing the gradient coefficients which are needed to retrospectively account for gradient non-linearity during reconstruction.

### Data processing

The DICOM data have been converted to NIfTI format using Statistical Parametric Mapping 12 (SPM) (ref. 19). Afterwards the volumes were bias field corrected with SPM utilizing a customized script. The graphical user interface of SPM offers only limited parameterization and therefore the correction was executed directly from MATLAB’s command line with adjusted parameters for 7 T data. Depending on the nature of the artifact induced by the bias field the parameters have to be adjusted, whereas FWHM and the regularization of the model are of major importance. If the bias field is of very low spatial frequency the FWHM should be broad and should be small for higher frequencies, respectively. If the data is heavily corrupted by the bias artifact, the regularization should be set lighter in order that the model has more flexibility. Following parameters were deviated compared to default: FWHM reduced from 60 to 15 mm, medium instead of light regularization, sampling distance reduced from 3 to 2 mm, and thorough cleanup instead of light cleanup. Subsequently, each volume has been rigidly re-oriented to have the anterior commissure (AC) and posterior commisure (PC) in the same axial slice and to separate the left and right hemisphere (mid-sagittal plane) by the AC-PC axis using ‘acpcdetect’ of the Automatic Registration Toolbox (ART)^20^. Afterwards the image’s origin was set to the anterior commissure manually to ensure best registration results based on a common origin. This was done as even with utmost care the positioning of the head inside the coil and manual placement of the FoV vary across different measurement sessions.

Another way to correct the bias field is to divide a MPRAGE by a gradient echo with the same sequence parameters, but without inversion recovery pulse^21^. This procedure additionally corrects for proton density and T_2_* contrast enabling mapping of T_1_ values and has been incorporated in the MP2RAGE sequence^22^. A drawback is the doubling of the scan time as another dataset with same repetition time (TR) has to be acquired. Furthermore, the SNR of the data needs to be high enough as noise of both images will be present in the divided image. Nonetheless, an appropriate gradient echo has been acquired in the first session alongside an MPRAGE. It has been included in the repository besides the ‘corrected by division’-volume, although this procedure was used on the first volume only.

Data which is acquired in one session is usually averaged after rigid registration, e.g., using SPM’s co-registration approach. This is feasible if no structural changes between the scans occurred. However, as our data was acquired over a time course of three month, the shape of the brain is not exactly identical between sessions. Depending on physiologically induced effects such as the level of hydration^23^ of the subject, the time of day^24^ etc. the brain may appear slightly different. Therefore, the average of the brain across sessions should not be generated by a rigid-body registration and determination of the arithmetic mean, as this may lead to ghost-like artifacts or blurring, e.g., around vessels, the ventricles, or the Dura mater. To handle this issue we chose an algorithm which is commonly used in building templates for atlas generation or group analysis. Using the diffeomorphic registration method^25^ incorporated in the Advanced Normalization Tools (ANTs, available at: https://github.com/stnava/ANTs) with its high accuracy and precision, we tried to generate the best possible average of the data within a reasonable amount of processing time. The flow chart to illustrate the entire processing pipeline is given in Fig. 1. The steps are described in detail with exactly specified command lines in the readme of the averaged dataset, which can be found in the repository. To average the single volumes we chose a script of ANTs for constructing a multivariate template. Using this script an initial template based on a rigid transformation is built first. Following that, the single volumes are registered to the initial template running consecutively a rigid, affine, and SyN (symmetric image normalization) transformation^26^ with cross-correlation as similarity metric. Parameters were specified in such way that the SyN registration method uses 20 iterations at a quarter resolution, 15 iterations at half resolution, and 5 iterations at full resolution. Subsequently, the arithmetic mean is calculated of all eight registered volumes. The averaged volume is then used as new template for another iteration of the same procedure. To build the final averaged template four iterations are performed in total. The computing time to get the final average was approximately four weeks using up to 24 threads @2,800 MHz with an average memory consumption of approximately 60 GB and a peak memory consumption of 90 GB.

### Code availability

All software used to reproduce the resulting averaged volume is freely available and shared by their respective owners.

The custom MATLAB script to run SPM12’s bias field correction with our 7 T parameters and a batchfile created with SPM12 are available within the repository. It utilizes unmodified functions of SPM12.

## Data Records

All data records listed in this section are available from the Dryad Digital Repository (Data Citation 1), with the exception of the scanner’s raw data. The raw data is hosted by the library of the Otto-von-Guericke University, Magdeburg (Data Citation 2).

### Averaged data

**Anatomical data** /derivatives/sub-01/averages/anat/sub-01_avg-0*_T1w.nii.gz

**File format** NIfTI, gzip compressed

**Additional information** /averages/readme.txt

**File format** Plain text

**Results** /derivatives/sub-01/results/results/avg-04/FreeSurfer_250um_to_1mm.tar.gz

**File format** tar gzip compressed

3D structural data after averaging all eight single measurements per iteration using ANTs with SyN registration method. Supplementary to the images in NIfTI format, a readme file containing additional information (e.g., command lines used to process data) is included in plain text. Results of processing the dataset with FreeSurfer v5.3 (downsampled to an isotropic resolution of 1 mm) are included as tar gzip compressed entire subject folder.

### Processed data

**Anatomical data** /derivatives/sub-01/ses-0*/anat/sub-01_ses-0*_run-0*_*.nii.gz

**File format** NIfTI, gzip compressed

**Additional information** /derivatives/readme.txt

**File format** Plain text

**Results** /derivatives/results/ses-0*/FreeSurfer_*mm.tar.gz

**File format** tar gzip compressed

**Scripts** /derivatives/scripts/

**File format** m-File and mat-file

Processed 3D T_1_-weighted structural data with an isotropic resolution of 250 μm, 500 μm and 1 mm. Processing consists of bias field correction (SPM12, using a custom script) and AC-PC alignment. Additionally, a division image of the T1w and PDw image of session 01 is included. Supplementary to the images in NIfTI format, a readme file containing additional information in plain text, the entire subject folder of FreeSurfer processing the 1 and 0.5mm data, and the used script to perform bias field correction (does require SPM12 to be installed) is included.

### Source data

**Anatomical data** /sourcedata/sub-01/ses-0*/anat/sub-01_ses-0*_run-0*_*.nii.gz

**File format** NIfTI, gzip compressed

**Additional information** /sourcedata/readme.txt

**File format** Plain text

**Sequence protocol** /sourcedata/sub-01/ses-0*/sequence_protocol.pdf

**File format** PDF

**Motion log** /sourcedata/sub-01/ses-0*/motion/sub-01_ses-0*_run-0*_*.log

**File format** Plain text

3D structural data of each single volume with T_1_- or PD-weighting. In session 01 to 05 data with a native isotropic resolution of 250 μm was acquired. In session 06 and 07 data with an isotropic resolution of 1 and 0.5 mm was acquired, respectively. The DICOM data was converted to NIfTI file format with additional extraction of metadata in JSON format using dicm2nii (http://www.mathworks.com/matlabcentral/fileexchange/42997). Supplementary to the images in NIfTI format, a readme file containing additional information in plain text, the exported sequence protocol as PDF file, and the motion log of the tracking system in plain text (only available for 250 μm data) is included.

### Raw data

**Image data** /sub-01_ses-0*_run-0*_*.h5

**File format** h5 (ISMRM raw data format)

Raw data in ISMRM raw data format of 3D T_1_-weighted MPRAGE and GRE data.

## Technical Validation

In order to assess the quality of the T_1_-weigthted images, FreeSurfer’s^29^ latest stable release (version 5.3) was used to perform a complete image segmentation and cortical surface reconstruction. The standard processing script ‘recon-all’ was run with default parameters which downsamples the data to 1 mm. The averaged 250 μm, the 500 μm, and 1 mm dataset was processed this way. FreeSurfer was able to perform the cortical surface reconstruction fully automatically and without any errors during processing. Afterwards FreeSurfer’s QA Tools v1.1 (https://surfer.nmr.mgh.harvard.edu/fswiki/QATools) have been used and no outliers were identified. Visual inspection shows good surface reconstructions, but manual intervention could improve results of all datasets. However, this is beyond the scope of the quality assessment, as manual intervention is needed in most cases. FreeSurfer’s entire subject folder of all three datasets has been included in the repository, consisting among other data of the image segmentation, surface reconstruction and results of the QA tools.

**Figure 4:**
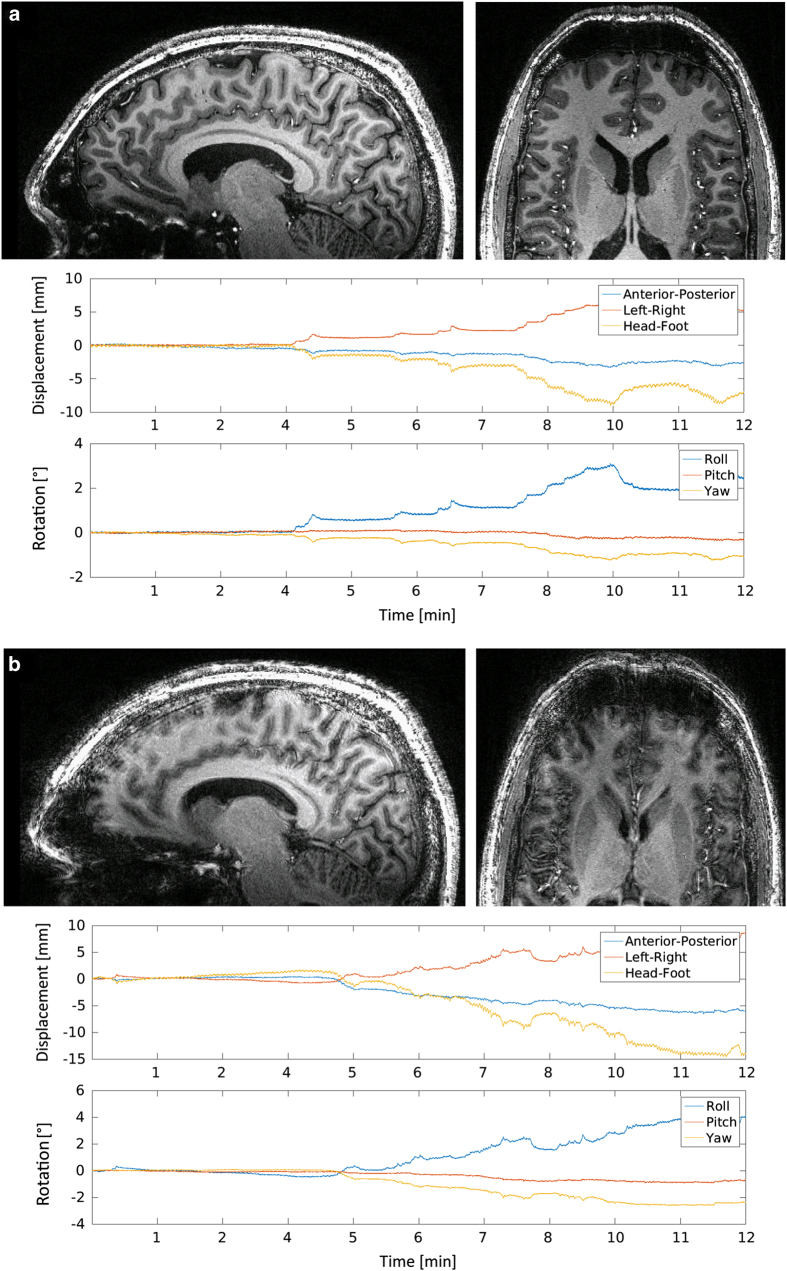
Illustration of the benefit using prospective motion correction. The same subject was scanned twice using a T_1_-weighted MPRAGE with an isotropic resolution of 450 μm. (**a**) shows a resulting image of the sagittal and axial view with motion correction turned on and **b** the according images with motion correction turned off. The displacement and rotation are quite similar between scans, allowing a good comparison.

Technical validation of the prospective motion correction procedure can be found in the publication of Maclaren *et al.*^17^ At present a study is being conducted in which the clinical benefit of the system is being evaluated. The results of a T_1_-weighted sample dataset with an isotropic resolution of 450 μm can be viewed in Fig. 4. The volume was acquired with the same MPRAGE sequence used to acquire the datasets of this study. The subject has been scanned twice, once with prospective motion correction turned on and once off within the same session. The subject was told to hold still and not to move voluntarily. Figure 4 illustrates the advantages of the system in case of large movements as the motion pattern of both measurements was very similar. In general, the displacement in head-foot direction induced by every heart beat is approximately on the order of 150 μm, whereas it amounts to approximately 350 μm per breathing cycle. As very little blurring can be perceived in the 250 μm datasets included in this study, it can assumed that involuntary minor rigid motion based on physiological effects, e.g., heart and respiratory cycle, are corrected also well.

## Usage Notes

The data can potentially be used to compare for resolution induced changes in quantitative image analysis, e.g., cortical thickness measurements or morphometric measures. This can be done by gradually downsampling the 250 μm dataset. Additionally, results can be compared against the included 500 μm and 1 mm data of the same subject.

By making the scanner’s raw data available we hope to encourage other groups to facilitate an improved reconstruction pipeline, e.g., using homodyne reconstruction instead of zero-filling for partial Fourier data to not degrade the image resolution. Furthermore, by utilizing compressed sensing methods to reconstruct the data, it may be noise filtered, increasing SNR and therefore CNR. This could serve a careful investigation of the resulting effects, such as potential blurring, removal of small structures or contrast changes. Other noise removal methods may be tested on single averages and can be compared to the averaged results of higher SNR.

The scanner’s raw data and information of the motion logs can potentially be used to de-correct the images, by reversing the position changes of the FoV per k-space line^30^.

## Additional Information

**How to cite this article:** Lüsebrink, F. *et al.* T_1_-weighted *in vivo* human whole brain MRI dataset with an ultrahigh isotropic resolution of 250 μm. *Sci. Data* 4:170032 doi: 10.1038/sdata.2017.32 (2017).

**Publisher’s note:** Springer Nature remains neutral with regard to jurisdictional claims in published maps and institutional affiliations.

## Supplementary Material



## Figures and Tables

**Table 1:** Imaging parameters of the MPRAGE sequence.

**Voxel size [μm]**	**TR [ms]**	**TE [ms]**	**TI [ms]**	**FA [°]**	**BW [Hz/px]**	**SPF**	**PPF**	**Matrix Size**	**ToA [min]**	**#**
250	3,580	2.41	1,210	5	440	6/8	8/8	880×880×640	≈53	8
500	2,740	3.24	1,050	5	130	6/8	8/8	416×416×352	≈19	1
1,000	2,500	1.91	1,050	5	250	8/8	8/8	256×256×176	≈11	1
For a full description we refer to the exported sequence protocol found in the repository.										

## References

[d1] *Dryad Digital Repository* Lüsebrink F. Sciarra A. Mattern H. Yakupov R. Speck C. 2016 http://dx.doi.org/10.5061/dryad.38s74

[d2] *Otto von Guericke University Library* Lüsebrink F. Sciarra A. Mattern H. Yakupov R. Speck C. 2017 http://dx.doi.org/10.24352/ub.ovgu-2017-001

