# Erratum: T_1_-weighted *in vivo* human whole brain MRI dataset with an ultrahigh isotropic resolution of 250 μm

**DOI:** 10.1038/sdata.2017.62

**Published:** 2017-05-09

**Authors:** Falk Lüsebrink, Alessandro Sciarra, Hendrik Mattern, Renat Yakupov, Oliver Speck

**Keywords:** Brain imaging, Brain, Imaging techniques, Magnetic resonance imaging

*Scientific Data* 4:170032 doi: 10.1038/sdata.2017.32 (2017); Published 14 March 2017; Updated 9 May 2017

The original HTML version of this Data Descriptor incorrectly included Oliver Speck’s name in the Data Citations as ‘Speck, C.’ instead of ‘Speck, O.’ This has now been corrected in the HTML version. The PDF version was correct at the time of publication, and has not been altered.

